# Sex Differences in Mitral Annular Calcification and the Clinical Implications

**DOI:** 10.3389/fcvm.2021.736040

**Published:** 2021-10-14

**Authors:** Jiwon Seo, Hyeonju Jeong, Iksung Cho, Geu-Ru Hong, Jong-Won Ha, Chi Young Shim

**Affiliations:** ^1^Division of Cardiology, Severance Cardiovascular Hospital, Yonsei University College of Medicine, Seoul, South Korea; ^2^Division of Cardiology, Department of Internal Medicine, Myongji Hospital, Hanyang University Medical Center, Seoul, South Korea

**Keywords:** mitral annular calcification (MAC), sex, echocardiography, mitral stenosis (MS), mitral valve (MV)

## Abstract

**Background:** Heterogeneous mechanisms may contribute to the occurrence of mitral annular calcification (MAC), however, little is known about the sex differences in MAC and the clinical implications of these differences. This study aimed to investigate clinical and imaging differences of MAC according to sex.

**Methods:** In total, 537 patients (221 men) with MAC were identified by transthoracic echocardiography at a single center from January 2012 to June 2016. Moderate-to-severe MAC was defined as calcification extent ≥120° of the mitral annulus. Significant functional mitral stenosis (MS) was defined as a transmitral mean diastolic pressure gradient ≥5 mmHg.

**Results:** Women more frequently had moderate-to-severe MAC and concomitant mitral regurgitation than men; however, significant functional MS was comparable between sexes. In the logistic regression analysis, old age, uncontrolled hypertension, end-stage renal disease (ESRD), and obstructive hypertrophic cardiomyopathy were significantly associated with moderate-to-severe MAC in women, whereas ESRD and moderate-to-severe aortic stenosis were in men. In the Cox regression analysis, significant functional MS was associated with all-cause death in both sexes, although an independent association was found in only women.

**Conclusion:** Women had more extended MAC than men. Significant functional MS was independently associated with unfavorable clinical outcomes in patients with MAC, which was more pronounced in women than in men.

## Introduction

Mitral annular calcification (MAC) is a chronic degenerative process in the fibrous base of the mitral valve (1). Early studies highlighted that MAC is associated with cardiovascular risk factors and is independently related to the occurrence of cardiovascular diseases such as stroke, coronary artery disease, and atrial fibrillation (2–4). These associations may explain why MAC is a progressive disease with features similar to atherosclerosis (5, 6). However, previous studies have shown that MAC is more frequent in women than in men, which cannot be explicated by the atherosclerosis paradigm (7, 8). This is probably related to other risk factors for MAC that differ according to sex, such as abnormal calcium-phosphate product or post-menopausal osteoporosis (9, 10). Therefore, it is assumed that there are differences according to sex regarding the mechanism, characteristics, and risk factors of MAC. Over the past decades, some studies have reported a sex difference in subjects with MAC. One study showed that larger calcium deposits more frequently occur in women than in men, based on a pathological evaluation of patients with MAC (9). Another study demonstrated that calcification activity in MAC was closely associated with female sex (11). Regardless of these accumulating results, the sex differences in the clinical and echocardiographic characteristics of subjects with MAC remain ambiguous. Thus, we aimed to investigate clinical and imaging differences of MAC according to sex. Additionally, we aimed to study sex-specific differences in the severity and prognosis of MAC.

## Materials and Methods

### Study Design and Population

In total, 537 patients with MAC were retrospectively identified by transthoracic echocardiography in a single tertiary institution (Yonsei University Cardiovascular Hospital, Seoul, Republic of Korea) from January 2012 to June 2016. Patients who had rheumatic mitral stenosis (MS), congenital mitral valve disease, an inadequate quality of echocardiographic data for assessment of MAC, and undergone mitral valve surgery were excluded. The study was approved by the local institutional review board (IRB no. 4-2017-0822) and complied with the Declaration of Helsinki. As this registry-based retrospective study and the data were analyzed anonymously, informed consent was not required from the study subjects.

### Echocardiography

**Figure 1:**
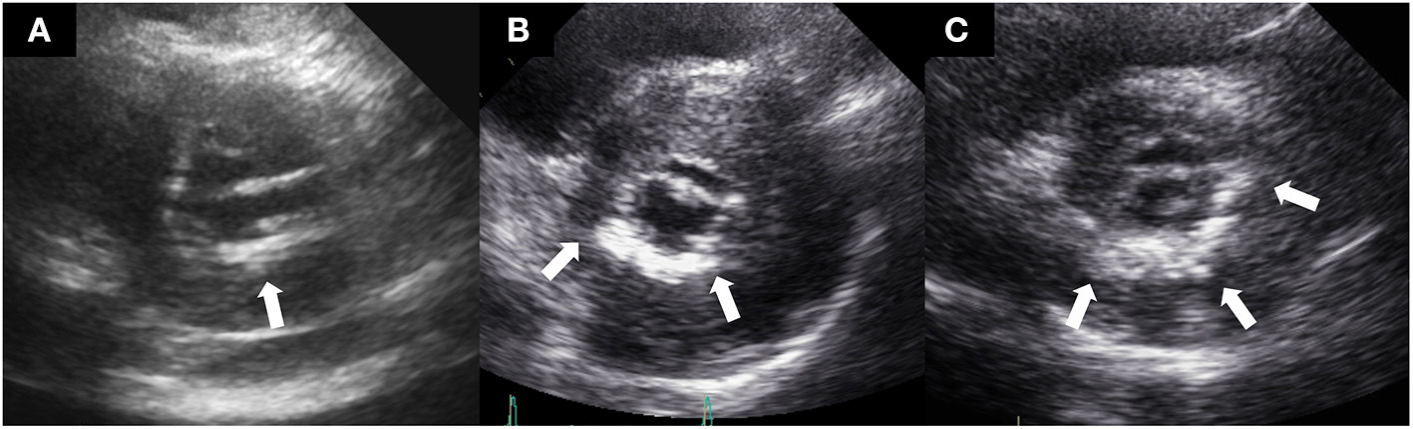
Representative cases of mild **(A)**, moderate **(B)**, and severe **(C)** mitral annular calcification.

Standard two-dimensional and Doppler echocardiography measurements were performed following the American Society of Echocardiography guidelines (12). MAC was defined as a thick and echo-dense area of the mitral annulus, occasionally extending to the mitral valve leaflets, as described in previous studies (3, 13). Comprehensive echocardiography reviews were performed to define functional and structural characteristics of the left ventricle and MAC (thickness, location, extent, and functional MS). To determine which annulus was mainly distributed with calcium, MAC was evaluated by parasternal long-axis, parasternal short-axis, apical 4-chamber, 3-chamber, and 2-chamber views. Severity of MAC was qualitatively determined in the parasternal short-axis view at the level of the mitral annulus as mild (focal, limited increase in echodensity within 120° of the mitral annulus), moderate (marked echodensity within 120–180° of the mitral annulus), or severe (marked echodensity at >180° of the mitral annulus) (14). MAC thickness was measured from the leading anterior to the trailing posterior edge at its greatest width. Figures 1A–C shows representative cases of mild, moderate, and severe MAC, respectively. The estimation of the diastolic pressure gradient was derived from the transmitral velocity flow curve using continuous wave Doppler echocardiography. Significant functional MS was defined as a transmitral mean diastolic pressure gradient ≥5 mmHg (15). In patients with atrial fibrillation, the mean gradient was calculated as the average of five cycles with the least variation of R–R intervals as close as possible to the normal heart rate.

### Clinical Data

Demographic, anthropometric, and laboratory data at the time of echocardiography were collected from patients' electrical medical records. All-cause death data were also collected from the electrical medical records. Diagnosis of hypertrophic cardiomyopathy (HCM) was made clinical criteria; maximal end-diastolic wall thickness of ≥15 mm anywhere in the left ventricle (LV), in the absence of another cause of hypertrophy. Uncontrolled hypertension was defined as systolic blood pressure ≥140 mmHg or diastolic blood pressure ≥90 mmHg in patients taking anti-hypertensive treatment. The severities of mitral regurgitation (MR) and aortic stenosis (AS) were determined according to the current guideline (16).

### Statistical Analysis

All continuous data are presented as mean ± standard deviation, and categorical data are expressed as number and percentage for each group. Univariable and multivariable linear regression analyses were performed to assess which factors were independent determinants of moderate to severe MAC. Univariate outcomes with a *p* < 0.1 were selected for inclusion in multivariable analysis. This linear regression analysis was performed separately in patients without end-stage renal disease (ESRD). Kaplan–Meier survival analysis was performed to evaluate the association of significant MS and all-cause death. Univariate and multivariate Cox regression analyses were performed to determine the risk factors of all-cause death. All tests were two-sided, and statistical significance was defined as *p* < 0.05. All statistical analyses were performed using R statistical software (version 3.6.0; R Foundation for Statistical Computing, Vienna, Austria).

## Results

### Baseline and Echocardiographic Characteristics

Among 537 patients, 316 were women, and the mean age was 74.7 ± 10.5 years. Women were significantly older and had a greater body mass index (BMI), higher systolic blood pressure, and higher pulse pressure than men. The prevalences of hypertension, diabetes, dyslipidemia, coronary artery disease, and AS were comparable between the sexes. Current smoking status, ESRD, and moderate-to-severe AS were more frequent in men than in women; however, HCM was more frequent in women than in men, as shown in Table 1. Women more frequently had MAC limited to the posterior annulus while combined anterior and posterior annular calcification was more frequently found in men. There were no differences in MAC thickness and significant MS between the sexes. MR was more frequently observed in women than in men; however, there was no significant difference in the prevalence of moderate or severe MR between the sexes. Women with MAC showed smaller LV dimensions, a lower e′ velocity, and a higher E/e′ than men with MAC (Table 2).

**Table 1:** Baseline characteristics.

	**Men (*n* = 221)**	**Women (*n* = 316)**	***P*-value**
Age, years	73 ± 11	76 ± 10	0.001
80 ≥ years old, *n* (%)	60 (27)	125 (40)	0.004
Body mass index, kg/m^2^	23.4 ± 3.0	24.4 ± 4.6	0.002
Systolic BP, mmHg	131 ± 22	137 ± 22	0.001
Diastolic BP, mmHg	71 ± 13	73 ± 13	0.181
Pulse pressure, mmHg	59 ± 19	64 ± 19	0.002
Uncontrolled hypertension, *n* (%)	81 (37)	145 (46)	0.225
Diabetes Mellitus, *n* (%)	75 (34)	99 (31)	0.525
Dyslipidemia, *n* (%)	110 (50)	163 (52)	0.680
Smoking, *n* (%)	112 (52)	21 (7)	<0.001
Coronary artery disease, *n* (%)	93 (42)	126 (40)	0.608
Atrial fibrillation, *n* (%)	48 (22)	69 (22)	>0.999
ESRD, *n* (%)	63 (29)	48 (15)	<0.001
Obstructive HCM, *n* (%)	2 (1)	15 (5)	0.024
Moderate or severe AS, *n* (%)	103 (47)	99 (31)	<0.001
Mitral valve prolapse, *n* (%)	2 (0.9)	3 (0.9)	>0.999
Osteoporosis, *n* (%)	15 (7)	73 (23)	<0.001

*BP, blood pressure; ESRD, end-stage renal disease; HCM, hypertrophic cardiomyopathy; AS, aortic stenosis*.

**Table 2:** Echocardiographic characteristics.

	**Men (*n* = 221)**	**Women (*n* = 316)**	***P*-value**
**MAC characteristics**			
Max thickness, cm	0.53 ± 0.28	0.52 ± 0.23	0.743
**Site**, ***n*** **(%)**			
Anterior MAC	2 (1)	5 (2)	0.768
Posterior MAC	166 (75)	264 (84)	0.022
Bilateral MAC	53 (24)	47 (15)	0.011
**Extents, n (%)**			
Mild (<120°)	155 (70)	167 (53)	<0.001
Moderate to severe (≥120°)	66 (30)	149 (47)	<0.001
Significant mitral stenosis, *n* (%)	23 (10)	34 (11)	>0.999
Mitral regurgitation, *n* (%)	79 (36)	152 (48)	0.006
No, *n* (%)	142 (64)	164 (52)	0.006
Mild, *n* (%)	59 (27)	111 (35)	0.049
Moderate, *n* (%)	14 (6)	35 (11)	0.084
Severe, *n* (%)	6 (3)	6 (2)	0.739
**Chamber characteristics**			
LVEDD, mm	51.3 ± 7.3	47.5 ± 6.4	<0.001
LVESD, mm	35.6 ± 8.6	31.8 ± 7.2	<0.001
LV mass index, g/m^2^	132.3 ± 46.8	123.6 ± 35.7	0.017
LA volume index, ml/m^2^	48.4 ± 18.7	49.2 ± 18.3	0.647
E velocity, m/s	0.91 ± 0.31	0.94 ± 0.31	0.234
e′ velocity, cm/s	4.6 ± 1.5	4.1 ± 1.4	<0.001
E/e′	21.4 ± 10.0	24.1 ± 9.4	0.003

*MAC, mitral annular calcification; LVEDD, left ventricular end-diastolic dimension; LVESD, left ventricular end-systolic dimension; LV, left ventricular; LA, left atrial; E, early diastolic mitral inflow; e′, early diastolic mitral tissue Doppler; E/e′, the ratio of early diastolic mitral inflow velocity to early diastolic mitral tissue Doppler velocity*.

### Factors Associated With Moderate to Severe Mitral Annular Calcification

In the multivariable logistic regression analysis, determinants of moderate-to-severe MAC were elderly age (≥80 years), female sex, ESRD, and obstructive HCM in the total study population. Only ESRD and moderate-to-severe AS were independently associated with moderate-to-severe MAC in men. On the contrary, elderly age, uncontrolled hypertension, ESRD, and obstructive HCM showed a significant relationship with moderate-to-severe MAC in women, as shown in Table 3. Similar associations were observed when excluding patients with ESRD (Supplementary Table 1).

**Table 3:** Associating factors with moderate to severe MAC.

	**Univariate**		**Multivariate**	
	**OR (95% CI)**	***P*-value**	**OR (95% CI)**	***P*-value**
**Total (*****n*** **=** **537)**				
Age	1.00 (0.99–1.01)	0.308		
80 ≥ years old	1.10 (1.01–1.20)	0.027	1.12 (1.02–1.22)	0.014
Female sex	1.19 (1.09–1.29)	<0.001	1.20 (1.10–1.30)	<0.001
Body mass index	1.00 (0.99–1.01)	0.491		
Current smoking	0.98 (0.81–1.19)	0.867		
Uncontrolled hypertension	1.07 (0.98–1.16)	0.129		
Diabetes mellitus	0.95 (0.87–1.04)	0.287		
Dyslipidemia	0.93 (0.85–1.01)	0.070	0.93 (0.86–1.01)	0.073
Coronary artery disease	0.99 (0.91–1.07)	0.764		
ESRD	1.14 (1.03–1.26)	0.012	1.22 (1.11–1.36)	<0.001
HCM obstructive	1.29 (1.02–1.63)	0.035	1.27 (1.01–1.60)	0.042
AS (≥ moderate)	0.99 (0.92–1.09)	0.960		
Osteoporosis	1.04 (0.93–1.16)	0.511		
**Men (*****n*** **=** **221)**				
Age	0.99 (0.99–1.00)	0.729		
80 ≥ years old	1.07 (0.94–1.23)	0.311	1.08 (0.94–1.24)	0.279
Body mass index	0.99 (0.97–1.01)	0.220		
Smoking	1.06 (0.94–1.20)	0.366		
Uncontrolled hypertension	0.94 (0.83–1.07)	0.333		
Diabetes mellitus	0.93 (0.82–1.06)	0.294		
Dyslipidemia	0.92 (0.81–1.03)	0.155		
CAD	1.04 (0.92–1.18)	0.510		
ESRD	1.17 (1.03–1.34)	0.019	1.21 (1.06–1.38)	0.005
HCM obstructive	0.74 (0.39–1.40)	0.356		
AS (≥ moderate)	1.16 (1.02–1.30)	0.019	1.16 (1.03–1.31)	0.014
Osteoporosis	0.90 (0.71–1.14)	0.390		
**Women (*****n*** **=** **316)**				
Age	1.00 (0.99–1.01)	0.371		
80 ≥ years old	1.08 (0.97–1.21)	0.164	1.14 (1.02–1.27)	0.026
Body mass index	1.01 (0.99–1.02)	0.412		
Smoking	0.87 (0.62–1.21)	0.401		
Uncontrolled hypertension	1.13 (1.01–1.26)	0.029	1.12 (1.00–1.25)	0.044
Diabetes mellitus	0.98 (0.87–1.10)	0.684		
Dyslipidemia	0.93 (0.83–1.04)	0.188		
CAD	0.96 (0.85–1.07)	0.434		
ESRD	1.20 (1.03–1.4)	0.021	1.25 (1.07–1.45)	0.006
HCM obstructive	1.32 (1.02–1.7)	0.038	1.42 (1.10–1.83)	0.008
Moderate or severe AS	0.93 (0.83–1.05)	0.257		
Osteoporosis	1.01 (0.89–1.15)	0.877		

*OR, odds ratio; ESRD, end stage renal disease; HCM, hypertrophic cardiomyopathy; AS, aortic stenosis*.

### Clinical Outcomes in Patients With Mitral Annular Calcification

**Figure 2:**
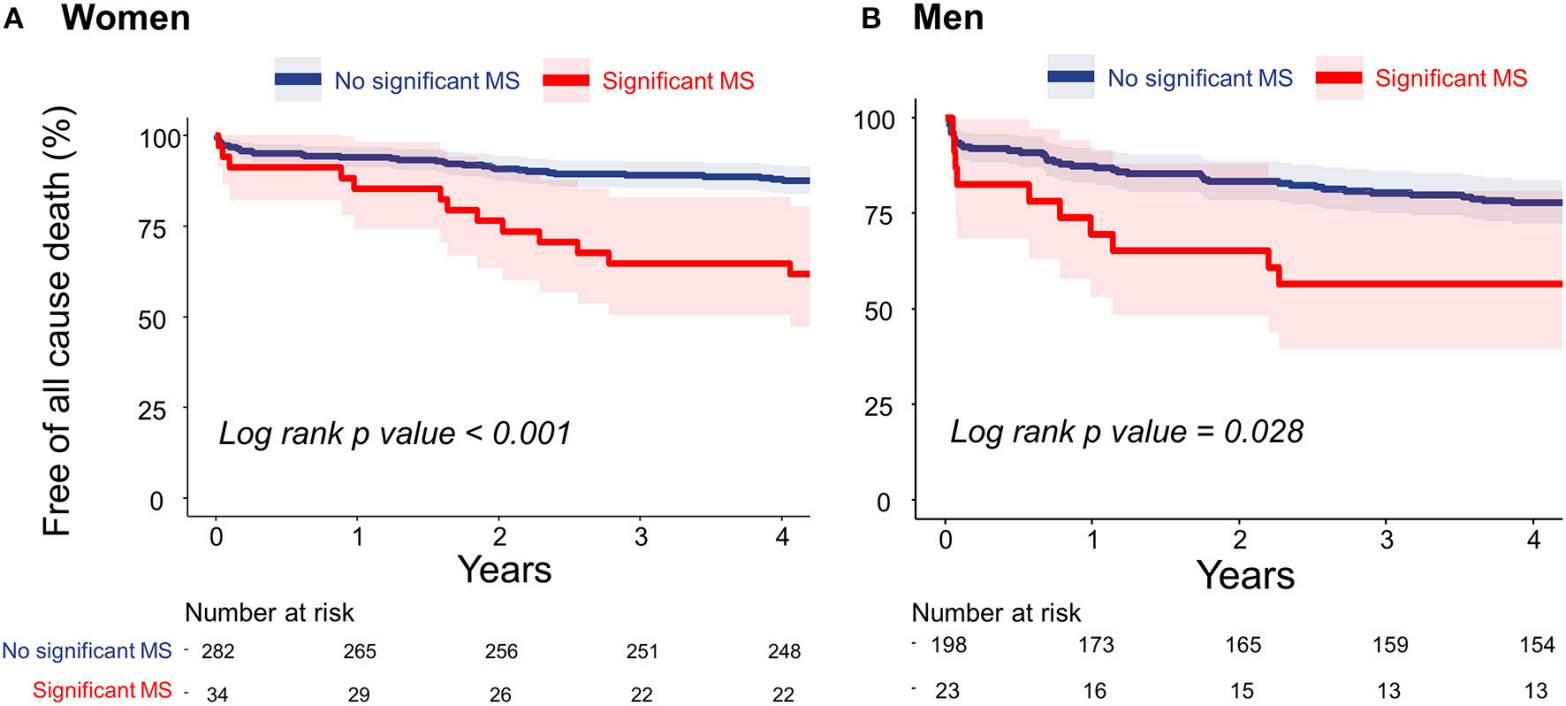
Cumulative incidences of all-cause death in women **(A)** and men **(B)** estimated using the Kaplan–Meier method. MS, mitral stenosis.

During the follow-up period (median, 5.3 years; interquartile range: 4.6–6.6 years), 106 deaths occurred. Figures 2A,B present the Kaplan–Meier curves to show the association between significant functional MS and all-cause death in both sexes. Patients who had significant functional MS had a higher probability of all-cause death than patients without significant MS, both in women and in men.

In the univariate Cox regression analysis, female sex and lower BMI were associated with a lower risk of mortality. Diabetes mellitus, ESRD, and significant MS were related to a higher risk of mortality. In the multivariate Cox regression analysis, older age, male sex, the presence of ESRD, and the presence of significant MS were independently associated with all-cause death (Table 4). When analyzing the data separately by sex, a lower BMI, ESRD, history of stroke, and significant MS were associated with mortality in men. However, only ESRD remained as a significant determinant of all-cause death in the multivariate model. In contrast, older age, diabetes mellitus, ESRD, and significant MS were significantly associated with mortality in the multivariate Cox regression model in women (Table 4).

**Table 4:** Associating factors with all-cause death in men and women.

	**Univariate**		**Multivariate**	
	**HR (95% CI)**	***P*-value**	**HR (95% CI)**	***P*-value**
**Total (*****n*** **=** **537)**				
Age	1.00 (0.99–1.02)	0.609		
Female Sex	0.56 (0.38–0.82)	0.003	0.65 (0.44–0.95)	0.027
BMI	0.93 (0.88–0.98)	0.004	0.95 (0.90–1.00)	0.061
Current smoking	0.96 (0.39–2.36)	0.933		
Uncontrolled hypertension	0.91 (0.62–1.33)	0.616		
Diabetes mellitus	1.51 (1.02–2.22)	0.038	1.38 (0.92–2.05)	0.110
Dyslipidemia	1.20 (0.82–1.76)	0.350		
Coronary artery disease	1.12 (0.76–1.64)	0.566		
ESRD	2.96 (2.01–4.36)	<0.001	2.04 (1.33–3.11)	0.001
Atrial fibrillation	1.23 (0.79–1.90)	0.354		
History of stroke	2.42 (1.30–4.52)	0.006	1.98 (1.04–3.74)	0.037
Moderate to severe MAC	1.02 (0.69–1.5)	0.927		
Moderate to severe MR	1.03 (0.57–1.88)	0.921		
Significant mitral stenosis	2.66 (1.67–4.22)	<0.001	2.05 (1.26–3.33)	0.004
HCM, obstructive	0.56 (0.14–2.25)	0.412		
Moderate or severe AS	0.92 (0.62–1.37)	0.685		
Osteoporosis	1.00 (0.99–1.02)	0.609		
**Men (*****n*** **=** **221)**				
Age	1.00 (0.98–1.02)	0.937	1.02 (0.99–1.05)	0.096
BMI	0.92 (0.85–1.01)	0.066	0.95 (0.87–1.04)	0.264
Current smoking	0.88 (0.32–2.44)	0.813		
Uncontrolled hypertension	0.89 (0.52–1.54)	0.682		
Diabetes mellitus	1.14 (0.66–1.94)	0.645		
Dyslipidemia	0.99 (0.59–1.67)	0.981		
Coronary artery disease	1.35 (0.81–2.28)	0.252		
ESRD	3.05 (1.81–5.13)	<0.001	3.11 (1.70–5.67)	<0.001
Atrial fibrillation	0.97 (0.51–1.83)	0.925		
History of stroke	2.46 (1.11–5.43)	0.026	1.85 (0.80–4.28)	0.149
Moderate to severe MAC	1.31 (0.76–2.25)	0.336		
Moderate to severe MR	0.55 (0.17–1.77)	0.317		
Significant mitral stenosis	2.12 (1.07–4.20)	0.031	1.59 (0.76–3.30)	0.218
HCM, obstructive	0.02 (0.01–9.99)	0.997		
Moderate or severe AS	1.04 (0.62–1.75)	0.875		
Osteoporosis	1.47 (0.59–3.69)	0.408		
**Women (*****n*** **=** **316)**				
Age	1.02 (0.99–1.06)	0.134	1.05 (1.01–1.08)	0.006
BMI	0.95 (0.88–1.00)	0.053	0.94 (0.88–1.01)	0.080
Current smoking	0.69 (0.09–4.97)	0.709		
Uncontrolled hypertension	1.02 (0.58–1.79)	0.943		
Diabetes mellitus	1.98 (1.13–3.47)	0.017	1.88 (1.05–3.37)	0.033
Dyslipidemia	1.50 (0.86–2.67)	0.166		
CAD	0.86 (0.48–1.54)	0.616		
ESRD	2.35 (1.26–4.36)	0.007	2.47 (1.22–5.02)	0.012
Atrial fibrillation	1.57 (0.86–2.89)	0.143		
History of stroke	2.08 (0.75–5.79)	0.160		
Moderate to severe MAC	0.99 (0.56–1.73)	0.965		
Moderate to severe MR	1.61 (0.78–3.31)	0.199		
Significant mitral stenosis	3.39 (1.80–6.40)	<0.001	2.89 (1.51–5.54)	0.001
HCM obstructive	0.84 (0.20–3.44)	0.803		
Moderate or severe AS	0.61 (0.31–1.19)	0.148		
Osteoporosis	1.51 (0.82–2.77)	0.186		

*HR, hazard ratio; BMI, body mass index; ESRD, end stage renal disease; MAC, mitral annular calcification; MR, mitral regurgitation; AS, aortic stenosis; HCM, hypertrophic cardiomyopathy*.

## Discussion

The main findings of the study were as follows: (1) Women had more extended MAC than men and had smaller LV dimensions, a lower e′ velocity, and a higher E/e′ than men; (2) older age, uncontrolled hypertension, ESRD, and obstructive HCM were associated with moderate-to-severe MAC in women, although only ESRD and moderate-to-severe AS were significantly associated with moderate-to-severe MAC in men; and (3) significant functional MS was independently associated with all-cause death, though this finding was only significant in women on multivariable analysis.

MAC is usually considered a benign condition where the incidence increases with age; it is often found incidentally *via* echocardiography or computed tomography. However, it has been revealed that MAC is independently associated with a higher risk of several cardiovascular diseases and mortality. In the Framingham Heart Study, MAC was related to an increased risk of cardiovascular disease and all-cause death (2). The northern Manhattan study, which was a multi-ethnic cohort study, reported that MAC was a strong and independent predictor of myocardial infarction and vascular death (3). Similarly, several cohort studies have shown that MAC is significantly associated with stoke (13, 17). Moreover, some characteristics of MAC had more association with stroke than conventional risk factors (4). Atrial fibrillation is also well-known to be associated with MAC in several studies (18–20). A *post-hoc* analysis from the Multi-Ethnic Study of Atherosclerosis (MESA) study showed that the accumulation of higher levels of MAC over time is associated with an increased risk for atrial fibrillation development and that important prognostic information regarding one's atrial fibrillation risk are conveyed by the presence and progression of MAC (18–20).

The risk factors of MAC are aging, atherosclerosis, conditions predisposing one to LV hypertrophy (e.g., hypertension, AS, or obstructive HCM), abnormal regulation of calcium-phosphorus products, and female sex (8). The reason for the association between women and MAC is explained by the loss of estrogen, which plays a role in atheroprotection (21, 22) and ectopic calcium deposits related to bone loss in post-menopausal women (23, 24). In addition to this explanation, the fact that women generally have a smaller heart and tend to develop greater hypertrophy in response to hypertension than men also may be reasons why MAC is more common and more severe in women than in men (25, 26). When stress on the mitral valve and annulus is increased due to various cardiac conditions, such as uncontrolled hypertension, AS, obstructive HCM, or mitral valve prolapse, calcific degeneration of the mitral valve can deteriorate gradually. This is associated with endothelial injury caused by mechanical stress, focal accumulation of oxidative stress, or a localized inflammatory process (8, 27). In our study, women had a smaller heart and worse diastolic function than men. Additionally, moderate-to-severe MAC was significantly associated with older age and uncontrolled hypertension only in women. Our results support that the mechanical force related to increased afterload on a relatively small ventricle may be important in the pathogenesis of MAC in women.

One interesting finding of our study is that the feature of MAC related to mortality was significant functional MS, but the extent of MAC was not. Early studies tended to focus on anatomical severity and its association with clinical outcomes. In the Framingham Heart Study, for each 1-mm increase in MAC thickness, the risk of incident all-cause death adjusted for relevant baseline risk factors increased by 9% (2). In the MESA study, MAC was a strong predictor for risk of cardiovascular events, and the risk was directly associated with anatomical MAC severity (3). Recently, MAC has been recognized as one of the important causes of MS. This kind of MS is called degenerative MS (DMS), and it can be progressive with an increase of calcium burden on the mitral valve (28, 29). Moreover, the prognosis of patients with DMS was poor; the 1 and 5-year survival rates were 78 and 47% in a retrospective single-center study (30). Bertrand et al. performed a retrospective study of echocardiography data from a large single institution to present the natural history of MAC. They showed the prognostic significance of the transmitral mean gradient for mortality in patients with MAC. Concomitant MR is associated with high mortality in only low gradient ranges (3–5 mmHg), indicating prognostic utility of the transmitral gradient in MAC regardless of MR severity (31). Our study also showed that only significant functional MS, defined as ≥5 mmHg of the transmitral pressure gradient, was independently associated with mortality. These discrepant results in each study were presumed to be due to different characteristics of study population. In the Framingham Heart Study and MESA study, clinical events were compared between participants with MAC and participants without MAC, whereas all patients in our study and in Bertrand et al.'s study had MAC. It is assumed that the relationship between the severity of MAC and prognosis was more emphasized when patients without MAC were included in the analysis.

### Limitations

The current study has several limitations. First, this study was retrospective in design, which causes an inherent limitation. Second, we reviewed echocardiography data retrospectively and identified MAC in patients from a single tertiary institution. Therefore, there may have been selection bias toward more severe MAC and greater risk of mortality. Third, evaluation of MAC using echocardiography may have limitations, especially in a retrospective setting. Depending on the quality of echocardiography, evaluation may be limited, and it is difficult to completely distinguish the boundary between MAC and normal tissue. Moreover, the MAC in the anterior annulus is often not well-imaged on the parasternal short-axis view. Further research combining 3D echocardiography or computed tomography may offer further insights. Fourth, only all-cause death was counted in the clinical events because it is difficult to determine the cause of death retrospectively and the follow-up period was different for each patient. However, since death is the most reliable and accurate clinical event, we assumed that it is an appropriate clinical event to identify which factors are associated with poor prognosis.

## Conclusions

There were sex differences in clinical and echocardiographic characteristics in patients with MAC. Women had more extended MAC than men. The mechanical force associated with increased afterload on a relatively small ventricle could be more dominant in the pathogenesis of MAC in women. Additionally, a significantly elevated transmitral pressure gradient was independently associated with all-cause death in patients with MAC, which was more pronounced in women than in men.

## Data Availability Statement

The raw data supporting the conclusions of this article will be made available by the authors, without undue reservation.

## Ethics Statement

The studies involving human participants were reviewed and approved by Yonsei University Institutional Review Board. The need for informed consent was waived by the Local Ethics Committee of the Yonsei University.

## Author Contributions

JS: data analysis, data collection, and drafting of the manuscript. HJ, J-WH, and G-RH: data collection and revising it critically for important intellectual content. IC: revising it critically for important intellectual content. CS: conception and design of study, drafting of the manuscript, and revising it critically for important intellectual content. All authors contributed to the article and approved the submitted version.

## Conflict of Interest

The authors declare that the research was conducted in the absence of any commercial or financial relationships that could be construed as a potential conflict of interest. The reviewer declared a past co-authorship with the several of the authors JS, HJ, IC, G-RH, J-WH, and CS to the handling editor.

## Publisher's Note

All claims expressed in this article are solely those of the authors and do not necessarily represent those of their affiliated organizations, or those of the publisher, the editors and the reviewers. Any product that may be evaluated in this article, or claim that may be made by its manufacturer, is not guaranteed or endorsed by the publisher.
