# Supplementary material for: Sex Differences in Mitral Annular Calcification and the Clinical Implications
Source: Front Cardiovasc Med. 2021 Oct 14;8:736040. doi: 10.3389/fcvm.2021.736040 (PMC8551453; doi:10.3389/fcvm.2021.736040)
Supplement: Supplementary file 1 [file Table_1.DOCX]

**Supplementary Data**

**Table S1. Associating factors with moderate to severe MAC in patients without end-stage renal disease**

|  | **Univariate** | | **Multivariate** | |
| --- | --- | --- | --- | --- |
|  | **OR (95% CI)** | **P-value** | **OR (95% CI)** | **P-value** |
| **Total (n=426)** |  |  |  |  |
| Age | **1.01 (1.01-1.02)** | **<0.001** | **1.01 (1.01-1.02)** | **<0.001** |
| Female sex | **1.21 (1.10-1.33)** | **<0.001** | **1.15 (1.05-1.26)** | **0.004** |
| Body mass index | 1.01 (0.99-1.02) | 0.204 |  |  |
| Current smoking | 0.90 (0.70-1.15) | 0.386 |  |  |
| Uncontrolled hypertension | **1.12 (1.02-1.23)** | **0.017** | **1.11 (1.01-1.21)** | **0.027** |
| Diabetes mellitus | 0.96 (0.86-1.06) | 0.378 |  |  |
| Dyslipidemia | 0.93 (0.85-1.02) | 0.147 |  |  |
| Coronary artery disease | 0.99 (0.91-1.10) | 0.987 |  |  |
| HCM obstructive | **1.39 (1.09-1.76)** | **0.008** | **1.40 (1.10-1.77)** | **0.006** |
| AS (≥ moderate) | 1.02 (0.93-1.12) | 0.714 |  |  |
| Osteoporosis | 1.02  (0.90-1.17) | 0.725 |  |  |
| **Men (n=158)** |  |  |  |  |
| Age | **1.01 (1.00-1.02)** | **0.008** | **1.01 (1.00-1.02)** | **0.020** |
| Body mass index | 0.99 (0.94-1.02) | 0.741 |  |  |
| Smoking | 1.03 (0.74-1.44) | 0.841 |  |  |
| Uncontrolled hypertension | 0.97 (0.84-1.13) | 0.723 |  |  |
| Diabetes mellitus | 0.93 (0.80-1.07) | 0.299 |  |  |
| Dyslipidemia | 0.93 (0.81-1.06) | 0.273 |  |  |
| CAD | 1.09 (0.95-1.25) | 0.225 |  |  |
| HCM obstructive | 0.78 (0.33-1.83) | 0.562 |  |  |
| AS (≥ moderate) | **1.19 (1.04-1.36)** | **0.013** | **1.16(1.01-1.32)** | **0.032** |
| Osteoporosis | 0.77  (0.55-1.07) | 0.117 |  |  |
| **Women (n=268)** |  |  |  |  |
| Age | 1.01  (1.00-1.02) | 0.007 | **1.01 (1.01-1.02)** | **0.002** |
| Body mass index | 1.01  (0.99-1.02) | 0.277 |  |  |
| Smoking | 0.82  (0.58-1.16) | 0.264 |  |  |
| Uncontrolled hypertension | 1.17 (1.04-1.32) | 0.009 | **1.18 (1.05-1.33)** | **0.006** |
| Diabetes mellitus | 0.97 (0.86-1.13) | 0.839 |  |  |
| Dyslipidemia | 0.95 (0.84-1.07) | 0.376 |  |  |
| CAD | 0.96 (0.85-1.08) | 0.491 |  |  |
| HCM obstructive | 1.36 (1.05-1.76) | 0.020 | **1.47 (1.14-1.90)** | **0.003** |
| AS (≥ moderate) | 0.97 (0.86-1.10) | 0.633 |  |  |
| Osteoporosis | 1.00 (0.86-1.16) | 0.995 |  |  |
| OR, odds ratio; ESRD, end stage renal disease; HCM, hypertrophic cardiomyopathy; AS, aortic stenosis | | | | |
